# Identification of Arabidopsis Candidate Genes in Response to Biotic and Abiotic Stresses Using Comparative Microarrays

**DOI:** 10.1371/journal.pone.0125666

**Published:** 2015-05-01

**Authors:** Arjun Sham, Khaled Moustafa, Salma Al-Ameri, Ahmed Al-Azzawi, Rabah Iratni, Synan AbuQamar

**Affiliations:** 1 Department of Biology, United Arab Emirates University, Al-Ain, United Arab Emirates; 2 Conservatoire National des Arts et Métiers (CNAM), Paris, France; Universidad Nacional Autonoma de Mexico, MEXICO

## Abstract

Plants have evolved with intricate mechanisms to cope with multiple environmental stresses. To adapt with biotic and abiotic stresses, plant responses involve changes at the cellular and molecular levels. The current study was designed to investigate the effects of combinations of different environmental stresses on the transcriptome level of Arabidopsis genome using public microarray databases. We investigated the role of cyclopentenones in mediating plant responses to environmental stress through TGA (TGACG motif-binding factor) transcription factor, independently from jasmonic acid. Candidate genes were identified by comparing plants inoculated with *Botrytis cinerea* or treated with heat, salt or osmotic stress with non-inoculated or non-treated tissues. About 2.5% heat-, 19% salinity- and 41% osmotic stress-induced genes were commonly upregulated by *B*. *cinerea*-treatment; and 7.6%, 19% and 48% of genes were commonly downregulated by *B*. *cinerea*-treatment, respectively. Our results indicate that plant responses to biotic and abiotic stresses are mediated by several common regulatory genes. Comparisons between transcriptome data from Arabidopsis stressed-plants support our hypothesis that some molecular and biological processes involved in biotic and abiotic stress response are conserved. Thirteen of the common regulated genes to abiotic and biotic stresses were studied in detail to determine their role in plant resistance to *B*. *cinerea*. Moreover, a T-DNA insertion mutant of the *Responsive to Dehydration* gene (*rd20*), encoding for a member of the caleosin (lipid surface protein) family, showed an enhanced sensitivity to *B*. *cinerea* infection and drought. Overall, the overlapping of plant responses to abiotic and biotic stresses, coupled with the sensitivity of the *rd20* mutant, may provide new interesting programs for increased plant resistance to multiple environmental stresses, and ultimately increases its chances to survive. Future research directions towards a better dissection of the potential crosstalk between *B*. *cinerea*, *abiotic stress*, and oxylipin signaling are of our particular interest.

## Introduction

Plants are immobile organisms convicted to face numerous environmental stresses during their lifetime. Biotic and abiotic stresses often occur suddenly and/or simultaneously; and, immediate plant responses are therefore critical to ensure cell survival [1]. A fundamental strategy for plants to adapt to environmental challenges imposed by biotic and abiotic threats is the modulation of gene expression. At the cellular level, plants tune gene expression along with their physiological needs to promote adaptation to short- as well as long-term environmental changes. Now, there is growing evidence that plants reprogram their responses under continuously changing environmental factors individually, or more frequently, in combination. Depending on the environmental conditions encountered, plants activate a specific program of gene expression [2]. The specificity of response is further controlled by a range of molecular mechanisms that “crosstalk” in a complex regulatory network, including transcription factors, kinase cascades, reactive oxygen species, heat shock factors and small RNAs that may interact with each other [3]. The interaction between biotic and abiotic stresses is orchestrated by hormone and non-hormone signaling pathways that may regulate one another positively or negatively. In response to biotic or abiotic stress, gene expression studies found that disease resistance-related genes in corn could be induced or repressed by abiotic stresses [4].

Several studies have identified the regulation of single genes in response to *B*. *cinerea* and abiotic stress. Arabidopsis *Botrytis Susceptible 1* (*BOS1*), *Botrytis-induced Kinase 1* (*BIK1*), *WRKY33* genes were previously identified [5–7]. In comparison with wild-type plants, the three mutants *bos1*, *bik1* and *wrky33* were extremely susceptible to *B*. *cinerea*. The MYB transcription factor, BOS1, plays a major role in plant defense response to *B*. *cinerea* that is regulated by jamonate (JA) [5]. The susceptibility of *bos1* mutant to *B*. *cinerea* was also linked to altered plant sensitivity to oxidative stress. *BIK1* gene, in turn, encodes a membrane-associated kinase protein in which *bik1* mutant showed high salicylate (SA) levels before and accumulated after *B*. *cinerea* inoculation [6]. While WRKY33 transcription factor showed a crosstalk between JA- and SA-regulated disease response pathways, both BIK1 and WRKY33 play an antagonistic role in plant defense as positive and negative regulators to resistance to *B*. *cinerea* and *Pseudomonas syringae pv tomato*, respectively [5, 6]. Efforts towards the identification of Arabidopsis *BOS1* interactors (*BOI*) and BIK1 regulators have led to uncover the function of some interactors and regulators in plant responses to pathogen infection and abiotic stress [8, 9]. Recently, the Arabidopsis mutation e*xpansin-like A2* (*EXLA2*) enhanced resistance to necrotrophic fungi, but caused hypersensitivity to salt and cold stresses [10]. Upon *B*. *cinerea* attack, an accumulation of cyclopentenones resulted in the repression of *EXLA2*; whereas *EXLA2* induction was dependent on abscisic acid (ABA) responses [10, 11].

The impact of an abiotic stress can also lead to increased resistance or susceptibility to a pathogen, or *vice versa*. The plant-parasitic nematode *Meloidogyne graminicola* reduced the damage of drought on rice (*Oryza sativa*) growth [3]. By contrast, drought-stressed sorghum (*Sorghum bicolor*) and common bean (*Phaseolus vulgaris*) showed increased susceptibility to the same fungus *Macrophomina phaseolina* [12, 13]. In Arabidopsis, drought-stressed plants showed severe susceptibility to the bacterial pathogen *P*. *syringae* [14]. On the other hand, in tomato (*Solanum lycopersicum*) and barley (*Hordeum vulgare*), it was found that increasing the tolerance level to drought, salt and osmotic stress also enhanced the resistance to *Blumeria graminis* and *B*. *cinerea* [15, 16]. These findings suggest that biotic and abiotic stresses may interact with each other positively or negatively and some microorganisms can thus be employed to efficiently enhance crop stress tolerance [17]. In fact, the combination of biotic and abiotic stresses activates the expression of unique and/or common sets of genes that are orchestrated by hormonal, mainly ABA, or non-hormonal pathways.

So far, limited attempts have been made to analyze gene expression changes in plants infected with pathogens and exposed to abiotic stresses. In Arabidopsis, a transcriptome profiling by microarray was performed in response to dehydration and the plant parasitic-nematode *Heterodera schachtii* [18]. Analysis of transcript profiles in Arabidopsis treated with flagellin, cold, heat, high light intensity and salt concentrations detects specific and shared responses between biotic and abiotic stresses and combinations of them [19]. A recent report on transcriptome analysis in Arabidopsis identified potential regulatory genes after infection with *B*. *cinerea* and treatments with cold, drought and oxidative stresses individually and in combination [20]. Here, we compare and analyse microarray data emanating from gene expression profiling in Arabidopsis in response to *B*. *cinerea* (biotic stress) and heat, salt and osmotic stresses (abiotic stresses). We analyzed plant responses to these stresses taken individually, and identified transcriptional regulatory networks at a single time point of gene expression. Arabidopsis plants were deliberately subjected to four individual stress treatments (one biotic and three abiotic stresses). In large, we combined the expression of *B*. *cinerea* upregulated genes (*BUG*s) with that of heat, salt or osmotic stresses; about 2.5%, 19% or 41% of the transcripts responded respectively, albeit the mode predicted from an individual stress treatment. With a minor increase in the fraction of the transcripts after combining *B*. *cinerea* downregulated genes (*BDG*s) with those of abiotic stress treatments, a transcriptional balance between plant responses to environmental stresses is suggested.

## Materials and Methods

### Plant growth and stress assays

We analyzed data from a previous study on *Arabidopsis* plants (ecotype Col-0) infected with *B*. *cinerea* [21]. In that study, the experimental conditions were conducted as follows: Five-week-old Arabidopsis plants were inoculated by placing four 5 μl drops of a 5 x 10^5^ spore mL^-1^ solution on each leaf. Control leaves were spotted with droplets of 24 g L^-1^ potato dextrose broth medium. Responses to *B*. *cinerea* infection were assayed at 18 and 48 hpi of adult leaves.

For the qRT-PCR and functional analyses, *B*. *cinerea* strain *BO5-10*, was grown on 2 x V8 agar (36% V8 juice, 0.2% CaCO3, 2% Bacto-agar). Fungal cultures were initiated by transferring pieces of agar containing mycelium to fresh 2 x V8 agar and incubated at 20–25°C. Collection of conidia from 10-day-old cultures and inoculation were carried out as previously described [6]. Disease assays were performed on whole plants or detached leaves (five-week-old plants) grown in soil were spray-inoculated or drop-inoculated (3 μL) with *B*. *cinerea* spore suspension (3x10^5^ spores mL^-1^) respectively, as described previously [10]. Control plants were sprayed with 1% Sabouraud maltose broth buffer using a Preval sprayer (Valve Corp., Yonkers, NY, USA). Plants were further kept under a sealed transparent cover to maintain high humidity in a growth chamber with 21°C day/18°C night temperature and a 12-h light/12-h dark photoperiod cycle. Responses to *B*. *cinerea* infection were assayed at 18 hpi of leaves, unless otherwise stated.

The drought sensitivity assay was performed on 3-week-old well-watered plants that were planted in soil. Seedlings were kept in a growth chamber under the same conditions mentioned above without watering (drought stress) for 10 days. Survival rates were scored 3 days after rewatering. Control plants were well-watered and kept under the same conditions.

### Identification of T-DNA insertion lines

T-DNA insertion lines were identified as described previously [22]. PCR primers were designed to the Arabidopsis genomic sequence flanking the T-DNA insertion site. These primers were used to analyze 12 sibling plants from each T-DNA line to confirm the T-DNA insertion cosegregated with the mutant phenotype. The primers were also used for genotyping individual lines within a segregating population to identify individuals homozygous for the insertion allele. A combination of one genomic primer plus a T-DNA insert primer was used to detect the insertion allele. Two genomic primers were used together to detect the wild-type allele. *rd20* (*SAIL_737_G01;* stock number N876376) was obtained from the Nottingham Arabidopsis Stock Centre (NASC, Nottingham, UK). The T-DNA insertion in the *rd20* mutant was confirmed by PCR using a T-DNA-specific primer (LB2, 5′-GCTTCCTATTATATCTTCCCAAATTACCAATACA-3′) and an *RD20*-specific primer (RP, 5′-AAGTACGGAACGATTTGGAGG-3′). Homozygous *rd20* mutant plants were identified by PCR using a pair of primers corresponding to sequences flanking the T-DNA insertion (LP, 5′-TTAACCGTTAGCGCGTATTTG-3′; RP).

### RNA extraction and expression analysis

RNA extraction and qRT-PCR expression analyses were performed as described previously [10]. The qRT-PCR was performed using gene-specific primers, with Arabidopsis *Actin2* (*AtActin2*) as an endogenous reference for normalization. Expression levels were calculated by the comparative cycle threshold method, and normalization to the control was performed as described [23]. Primer sequences are found in S1 Table.

### Statistical analysis

For each sample, three technical replicates of the qRT-PCR assay were used with a minimum of three biological replicates. Results were expressed as means ± standard deviation (SD) of the number of experiments. A Student’s *t*-test for the values was performed at *P* < 0.05.

Data of *B*. *cinerea* growth in inoculated plants represent the mean ± SD from a minimum of 16 plants. Data of drought sensitivity assay performed on plants represent the mean ± SD (n = 12). Analysis of variance and Duncan’s multiple range test were performed to determine the statistical significance [24]. Mean values followed by an asterisk are significantly different from the corresponding control (*P* < 0.05). All experiments were carried out in triplicate with similar results.

### Heat, salinity and osmotic stress treatments

We analyzed data from a previous study on the responses of Arabidopsis to various stress conditions [21]. In that study, seeds (ecotype Col-0) were surface-sterilized by treating them sequentially in 70% ethanol for 2 min, then 30% Clorox solution containing 0.01% Tween for 10 min, and rinsed several times in sterile water. Seeds were plated on media containing the Murashige and Skoog (MS) growth medium, 2% sucrose, 0.7% (w/v) purified agar, unless otherwise stated. Plates were kept at 4°C for 48h to synchronize germination, transferred to growth chambers with fluorescent lights, and maintained under the environmental conditions as described in [25] with some modifications.

For the heat stress experiment, sixteen-day-old seedlings were treated with either liquid-MS media at 25°C (control) or exposed to 38°C for 24h. For the salt and osmotic stress experiments, sixteen-day-old plants were treated with either liquid-MS media (control) or stressed by 150 mM NaCl (salt stress) or 300 mM Mannitol (osmotic stress) for 24h. All treatments and preparations were done on the same batch of seedlings, as described in [21].

### Data source and analysis

Raw microarray datasets were downloaded from NASCArrays [affy.arabidopsis.info/link_to_iplant.shtml] [21] for each stress. Data of “shoots” class were analyzed using R Statistical Computing [26], which uses Affy and MAS5 packages for data normalization. Affy computes the probe set signal intensity; whereas MAS5 computes the detection calls of each probe ID displayed as Present (P), Absent (A) and Marginal (M). The reference numbers are: control (for all abiotic stresses), NASCArrays-137; osmotic stress, NASCArrays-139; salt stress, NASCArrays- 140; heat stress; NASCArrays-146; and *B*. *cinerea*, NASCArrays-167 (including non-inoculated control). The number of tested samples (n) for each treatment is 8 (control; and heat stress), 6 (salt; and osmotic stresses), and 2 (*B*. *cinerea* and its control); with 22810 genes per array. Log_2_-transformed expression level data were used to generate scatter plots to detect the effect of *B*. *cinerea* infection at 18 hpi or abiotic stress treatment at 24 hours post-treatment (hpt) on plant gene expression. Comparisons of three replicates for each set of experiment were performed. In all samples, probes with expression labelled as ‘A’ or ‘M’ across all samples were removed from the dataset. At the tested time point, the overall gene expression difference between control (non-treated/non-inoculated) and treated/inoculated samples was determined by pairwise comparison. The normalized-fold change value for each gene was calculated by dividing the expression level of a treated/inoculated sample by the expression level of a non-treated/non-inoculated sample. A twofold or half-fold (unless otherwise stated) difference in expression level between treated/inoculated and non-treated/non-inoculated samples at *P* < 0.05 was set as the threshold for considering a gene to be up- or down-regulated, respectively. The cutoffs of the fold change were chosen to filter false positives and to compare our data analyses with those in the microarray literatures. All genes across the microarrays data were identified using the Arabidopsis Information Resources (TAIR; www.arabidopsis.org). We used microarrays data of treated seedlings with *B*. *cinerea*, cold, drought and oxidative stress as described [20]; and 12-oxo-phytodienoic acid (OPDA) and phytoprostane A_1_ (PPA_1_) as previously described [11, 27].

## Results

### Identification of differentially expressed genes to abiotic stresses

**Fig 1:**
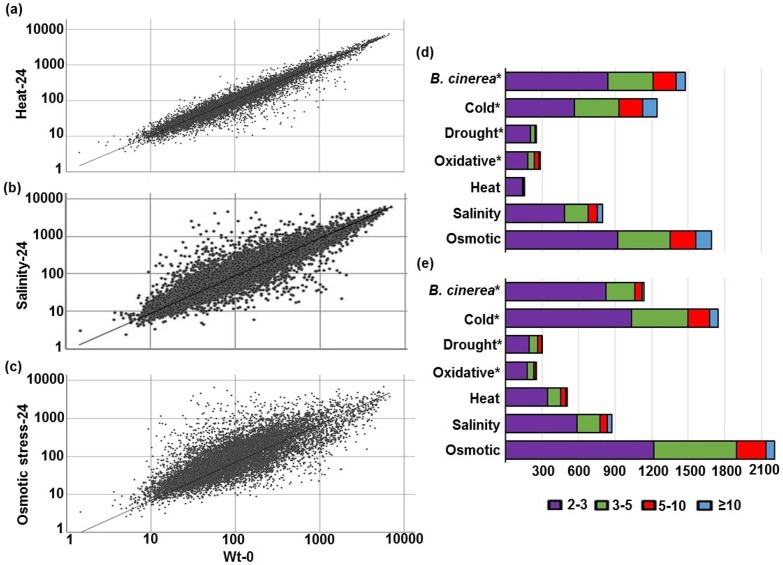
Comparisons of gene expression in Arabidopsis plants under biotic and abiotic stress conditions. Normalized expression values for each probe set in stressed plants with heat (A); salinity (B); or osmotic stress (C) at 24 hpt is plotted on the Y-axis. In (A-C), the value in wild-type plants sampled before the abiotic stress treatment (0 hpt; WT-0) is plotted on the X-axis. Number and the level of transcripts identified as upregulated (D), or downregulated (E) genes in Arabidopsis stressed plants. In (D-E), the treatment of the tested abiotic stress is plotted on the Y-axis; the number of differentially expressed genes is plotted on the X-axis. Columns with different colors show the fold change of corresponding differentially expressed genes. *Results were obtained from [20]. hpt, hours post treatment.

In this study, we aimed to identify components of the regulatory networks involved in Arabidopsis responses to *B*. *cinerea* infection and abiotic stresses (heat, salinity and osmotic stress). A full microarray-based analysis of Arabidopsis whole-genome Affymetrix gene chip (ATH1) representing approximately 25,000 genes was downloaded from NASC [21] to identify regulated genes by *B*. *cinerea* infection and the abiotic stress. To determine up- and down-regulated genes in Arabidopsis seedlings exposed to heat; salt; and osmotic stress treatments at 24 hpt, we first identified differentially regulated genes by comparing the expression profile of untreated- (control) or treated tissues in Arabidopsis wild-type plants (Fig 1A–1C). The transcript level for each gene before and after the treatment with heat, salinity or osmotic stress was assessed and compared. Genes with expression changes of more than twofold or less than half-fold (*P* < 0.05) were defined as significantly stress up- or down-regulated genes, respectively. The complete list of induced and repressed genes to heat, salinity or osmotic stresses is available (S2 Table). We also investigated whether the accumulated transcripts were functionally involved in stress response and defense. Based on the Gene Onology (GO) annotation, we classified the differentially expressed genes according to their biological and molecular activities, and cellular components. Our analysis showed that the differentially expressed genes in Arabidopsis seedlings under heat, salinity and osmotic stress conditions were majorly grouped as responsive to biotic and abiotic stimuli/stresses, electron transport, cell organization and development, and other biological processes (S1 Fig). The stress up-regulated genes encode for receptors, transcription factors, transporters, and enzymes (*i*.*e*. hydrolyases, kinases, transferases) corresponding to various cellular activities, mainly localized in the cell wall, Golgi apparatus, plastids and plasma membrane, suggesting an involvement of extracellular and intracellular components in plant response/defense to abiotic stress constraints.

*BUG*s and *BDG*s have been previously identified based on their transcriptional levels in response to *B*. *cinerea* infection at 18 hpi and differentially expressed genes were also identified in response to cold, drought and oxidative stress [20]. Data were analyzed to have a complete set of up- and down-regulated genes of major abiotic stress compared with those of *BUG*s or *BDG*s. Our microarray analysis showed there were 1498 genes considered as *BUG*s and 1138 genes considered as *BDG*s (Fig 1D and 1E). In addition, the gene expression levels under heat, salinity and osmotic stress treatments were altered for 660, 1649 and 3905 transcripts, respectively from which 153, 799 and 1695 genes were stress-induced genes. In most cases, there were more repressed than induced genes except for *B*. *cinerea* treatment. The average fold changes of differentially expressed genes ranged from 2–3 folds, though some genes showed 10-fold or more (S2 Table). It is worth mentioning that the number of genes involved in *B*. *cinerea*, cold, salinity and osmotic stress responses seems to be greater than those involved in drought, heat and oxidative stress responses (Fig 1D and 1E). This might be due to the fact that Arabidopsis is naturally more adapted to drought, heat and oxidative stress than to other environmental stress conditions.

### Common differentially expressed genes by *B*. *cinerea* and major abiotic stresses

**Fig 2:**
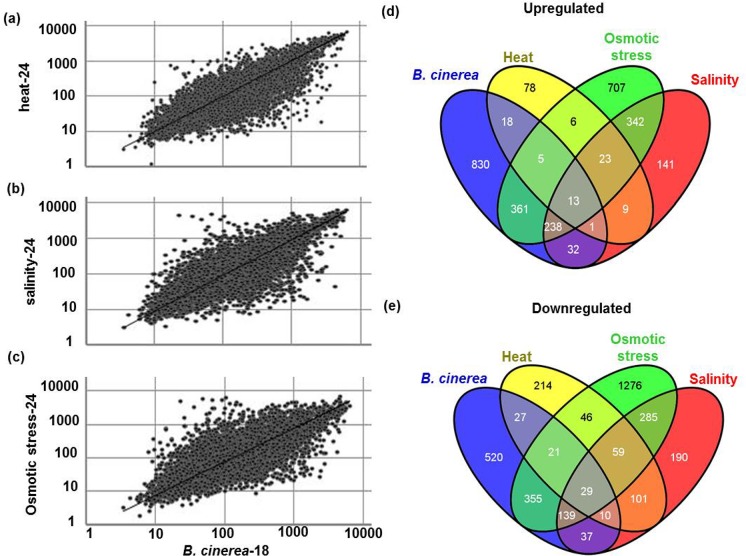
Scatter-plot comparisons of gene expression and number of *BUG*s and *BDG*s affected by abiotic stress. Normalized expression value for each probe set in wild-type plants infected with *B*. *cinerea* at 18 hpi (*B*. *cinerea*-18) is plotted on the X-axis; the value in stressed plants with heat (A); salinity (B); or osmotic stress (C) at 24 hpt is plotted on the Y-axis. The Venn diagram shows the number of *BUG*s (D); and *BDG*s (E) at 18 hpi that are also affected by heat, salinity and osmotic stress at 24 hpt. hpi/hpt, hours post inoculation/treatment.

To compare normalized transcriptional levels of genes identified as *B*. *cinerea*- and abiotic stress-regulated genes, scatter plots were constructed on the correlating genes between *B*. *cinerea* [20] and heat, salinity or osmotic stress (Fig 2A–2C). Similar patterns of gene expression levels were illustrated between Arabidopsis plants infected with *B*. *cinerea* at 18 hpi, and cold, drought or oxidative stress at 24 hpt [20]. Venn diagrams displayed that 37 genes were commonly upregulated by *B*. *cinerea* inoculation and heat treatment; whereas 87 were downregulated by the same stresses, representing 2.5% and 7.6% of the genes that were upregulated and downregulated by *B*. *cinerea*, respectively (Table 1).

**Table 1:** Regulation of *B*. *cinerea*-regulated genes by different stimuli.

Treatment	Co-upregulated genes		Co-downregulated genes	
	N^o^ of genes	Percentage^a^	N^o^ of genes	Percentage
Cold^b^	373	24.9	377	33.1
Drought^b^	92	6.1	77	6.8
Oxidative stress^b^	176	11.7	63	5.5
Heat	37	2.5	87	7.6
Salinity	284	19.0	215	18.9
Osmotic stress	618	41.2	546	47.8
All stresses	3	0.2	12	1.1

Shown are percentages of *BUG*s and *BDG*s (at least twofold) that were also at least twofold increased or decreased by the abiotic stress listed above.

^a^Percentage = N^o^ of up- or down-regulated genes of the abiotic stress/N^o^ of *BUG*s (1498 genes) or *BDG*s (1138 genes). *BUG*s and *BDG*s were obtained from [20].

^b^Results were obtained from [20].

The diagram also demonstrated that 284 genes were induced by both *B*. *cinerea* and salinity and 215 were repressed by these stresses (Fig 2D and 2E), each corresponding to 19% of either *BUG*s or *BDG*s (Table 1). About 40–50% of the identified *B*. *cinerea*-regulated genes were also regulated by osmotic stress. The list of the overlapping up- and down-regulated genes with distinct responses to *B*. *cinerea* and abiotic stress treatment is shown in S3 Table. To compare the co-regulation between *B*. *cinerea* and other classes of major abiotic stress from those subjected here, the analysis was extended to include *B*. *cinerea*-regulated genes with cold, drought and oxidative stresses that were previously identified (Table 1). Among the induced genes, 251 were shared in *B*. *cinerea*, salinity and osmotic stress treatments, while 18 and 14 were commonly upregulated by *B*. *cinerea*/heat/osmotic stress and *B*. *cinerea*/heat/salinity treatments, respectively (Fig 2D). Likewise, a common downregulation of genes was observed between *B*. *cinerea* and abiotic stress treatments where fifty and 39 of the shared genes showed downregulation by *B*. *cinerea*/heat/osmotic stress and *B*. *cinerea*/heat/salinity treatments, respectively (Fig 2E), while 13 induced genes and 29 repressed were common between all tested biotic and abiotic stresses (Fig 2D and 2E). When we compared with cold, drought and oxidative stresses data, we found that 15 genes were commonly responsive; three genes showed common induction with *BUG*s and 12 genes showed common repressions with *BDG*s (Table 1). Taken together, these findings suggest an overlap between *B*. *cinerea*, salinity and osmotic stress.

**Fig 3:**
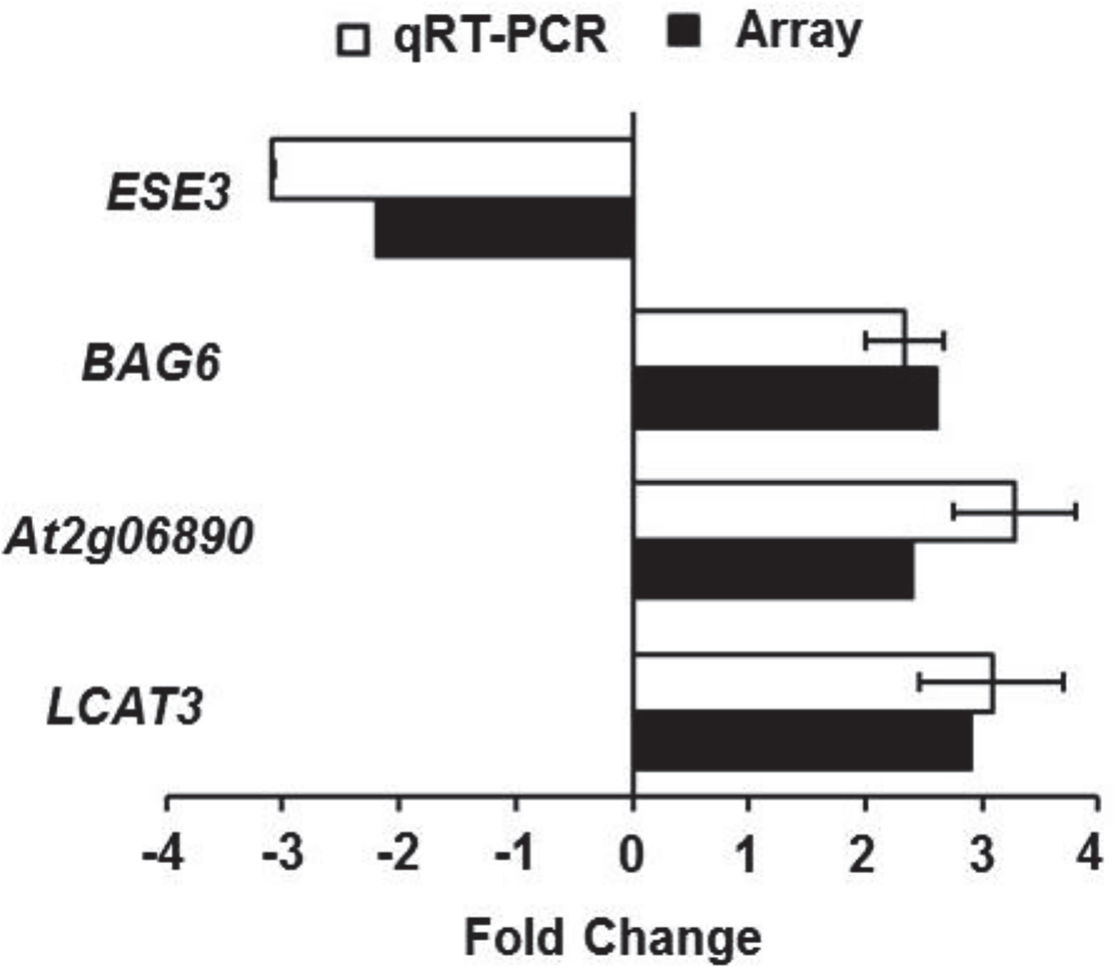
Comparison of values obtained for differential expression using qRT-PCR and microarrays. Relative expression levels obtained through qRT-PCR were compared with microarray expression levels (NASCArrays) for selected common *B*. *cinerea*- and abiotic stress-upregulated or-downregulated genes after infection with *B*. *cinerea* at 18 hpi. Expression of *B*. *cinerea*-induced or-repressed genes was quantitated relative to control conditions (no infection), and corrected for expression of the control β-actin gene. Microarray expression data were obtained from Tables 1 and 2. Error bars for qRT-PCR values are the standard deviations (n ≥ 3). hpi, hours post inoculation; *At Actin2*, Arabidopsis *Actin2* gene.

We looked carefully at the common up- and down-regulated members expressed by *B*. *cinerea*, heat, salinity and osmotic stress; and we found that some genes were frequently expressed to combined types. For example, the common *B*. *cinerea*/heat/salinity/osmotic stress-induced *At5g22860* and *At2g33380* (*RD20*), and the repressed *At5g25190* (Table 2) were previously identified as common respondents to *B*. *cinerea*, cold, drought and oxidative stress [20]. This suggests that although some genes were quite specific to *B*. *cinerea*, heat, salinity and osmotic stress; others showed general regulation to biotic and abiotic stresses. We also assessed a selected number of commonly differentiated expressed genes to *B*. *cinerea* infection using quantitative real time-PCR (qRT-PCR) to validate the microarray analysis. Relative gene expression changes measured by qRT-PCR in *B*. *cinerea*-infected leaves at 18 hpi were compared with Arabidopsis microarrays’ data. Similar transcript patterns for the tested genes, *ESE3*, *BAG6*, *LCAT3* and *At2g06890* were observed in the two approaches (qRT-PCR and microarrays) (Fig 3). We believe that the overlapping genes are not only functional in signal transduction pathways, mediated by phytohormones, but also in biotic and abiotic stress pathways that share many overlapping steps in non-enzymatic free radical-catalyzed pathway.

**Table 2:** Changes in expression of up-/down-regulated genes encoding putative proteins during *B*. *cinerea* infection and heat, salinity, and osmotic stress treatments in wild-type Arabidopsis plants.

Gene locus	Gene family	Probe set	*B*.*cinerea* ^a^ ^,^ ^b^	Abiotic stress^a^		
				Heat	Salinity	Osmotic stress
*At5g22860*	serine carboxypeptidase S28	249860	6.511	2.222	3.116	12.929
*At5g06190*	Unknown	250722	2.241	2.133	3.335	3.757
*At4g13800*	permease-related	254683	2.487	2.425	3.214	12.075
*At4g12910*	SCPL20	254791	3.236	2.070	2.909	2.735
*At2g33380*	RD20	255795	5.153	2.360	5.936	26.651
*At3g14067*	subtilase	256997	2.271	2.166	2.684	6.830
*At3g03310*	LCAT3	259057	2.88	2.38	5.18	17.57
*At3g05030*	NHX2	259081	2.627	3.144	3.396	4.889
*At1g70900*	Unknown	262313	2.10	2.01	2.83	4.92
*At2g42540*	COR15A	263497	7.40	2.88	88.16	102.16
*At2g06890*	transposable element gene	266214	2.43	2.40	2.18	2.44
*At2g46240*	BAG6	266590	2.631	2.023	56.992	3.703
*At2g39250*	SNZ	267010	2.413	2.432	4.054	11.476
*At5g25190*	ESE3	246932	-2.18	-3.85	-8.93	-5.73
*At5g49450*	BZIP1	248606	-2.94	-5.76	-2.47	-8.42
*At5g48430*	aspartyl protease/Pepsin A30	248703	-2.08	-2.28	-4.65	-3.80
*At5g41080*	GDPD2	249337	-2.19	-11.50	-3.33	-8.52
*At5g39580*	Peroxidase	249459	-6.16	-9.85	-7.38	-11.29
*At5g19120*	aspartyl protease/Pepsin A20	249923	-2.08	-5.61	-14.62	-27.66
*At5g05440*	PYL5/RCAR8	250777	-2.24	-8.22	-15.34	-11.26
*At3g50560*	SDR	252167	-5.21	-2.15	-6.98	-5.91
*At3g50060*	MYB77	252193	-3.01	-4.63	-5.27	-2.43
*At3g46280*	protein kinase-related	252511	-10.92	-15.38	-5.26	-25.77
*At4g21870*	HSP26.5-P	254384	-2.18	-3.06	-9.16	-7.77
*At4g12470*	protease inhibitor (AZI1)	254818	-4.07	-13.71	-14.99	-14.45
*At4g01250*	WRKY22	255568	-2.15	-5.63	-3.75	-4.13
*At4g01720*	WRKY47	255596	-2.58	-2.52	-3.12	-4.49
*At3g14770*	nodulin MtN3	256548	-3.54	-2.60	-2.56	-3.25
*At3g15950*	TSA1-LIKE (NAI2)	257798	-23.49	-2.54	-2.69	-3.33
*At3g16460*	jacalin lectin	259327	-16.43	-2.29	-4.22	-7.73
*At1g28010*	ABCB14/MDR12/PGP14	259579	-2.80	-2.89	-3.29	-3.49
*At1g21910*	DREB26	260856	-5.69	-14.79	-22.89	-3.68
*At1g19610*	PDF1.4/LCR78	261135	-4.85	-5.36	-5.36	-7.44
*At1g21830*	Unknown	262488	-2.72	-2.92	-3.11	-3.56
*At1g14890*	Invertase/pectinesterase inhibitor	262844	-2.82	-2.05	-2.37	-3.59
*At1g23870*	TPS9	263019	-3.45	-3.50	-2.54	-4.46
*At1g54740*	Unknown	264238	-2.60	-3.62	-3.10	-3.75
*At1g76930*	EXT4	264960	-2.30	-7.08	-3.18	-4.63
*At1g24530*	transducin /WD-40 repeat	265028	-4.69	-6.35	-5.48	-4.05
*At2g20670*	Unknown	265387	-4.33	-15.19	-3.60	-17.86
*At2g26980*	CIPK3	266313	-3.18	-2.10	-2.75	-3.84
*At2g40000*	HSPRO2	267357	-2.16	-4.50	-2.63	-8.24

^**a**^ Fold change in expression for each gene was calculated by dividing the expression level of a *B*. *cinerea*-infected or abiotic stress-treated sample by the expression level of a non-infected or non-treated sample, respectively. A twofold difference in expression level between *B*. *cinerea*-inoculated and non-inoculated or abiotic stress-treated and non-treated samples was set as the threshold for considering a gene to be *B*. *cinerea*- or abiotic stress up-/down-regulated gene *(P* < 0.05).

^**b**^ *B*. *cinerea* up-/down-regulated genes data were obtained from [20].

-, downregulation.

### Phenotypic analysis of T-DNA insertion mutants of overlapping genes to *B*. *cinerea* infection

To determine the function of the overlapping genes in responses to biotic and abiotic stress treatments (Table 1), we isolated mutants in selected regulated genes encoding putative regulatory proteins. T-DNA insertion lines for these genes were identified from the Syngenta Arabidopsis Insertion Collection (SAIL), the Salk Institute (SALK) T-DNA collection and the Plant Breeding Research GABI-Kat [22]; obtained from the NASC. Lines with homozygous insertions corresponding to 13 genes were isolated. The T-DNA insertion mutant lines were then challenged with *B*. *cinerea* as described [10], and a summary of the disease assay results is presented in Table 3. Most of the T-DNA mutant alleles had no detectable effect on the resistance phenotype, including insertions in *NHX2*, *SNZ*, *BZIP1*, *GDPD2*, *SDR*, *MYB77*, *WRKY77*, *CIPK3*, *At5g19120*, *At5g48430*, and *At4g21870* (Table 3).

**Table 3:** Phenotypic analysis of T-DNA insertion alleles of common-regulated genes in response to *B*. *cinerea*.

AGI number (probe set) ^a^	Protein/gene	Insertion site	SAIL/SALK ID (stock number)	Phenotype^b^
*At2g33380 (*255795*)*	RD20	Exon	SAIL_737_G01 (N876376)	S
*At3g05030 (*259081*)*	NHX2	Exon	SALK_039611 (N657915)	Wt
*At2g39250 (*267010*)*	SNZ	5’-UTR	SALK_030031 (N668027)	Wt
*At5g49450 (*248606*)*	BZIP1	Exon	SALK_069489 (660942)	Wt
*At5g48430 (*248703*)*	aspartyl protease/Pepsin A30	Promoter	SALK_128791 (N684580)	Wt
*At5g41080 (*249337*)*	GDPD2	Promoter	SALK_047427 (N653183)	Wt
*At5g19120 (*249923*)*	aspartyl protease/Pepsin A20	Exon	GABI_023B01 (N402125)	Wt
*At3g50560 (*252167*)*	SDR	Exon	SAIL_424_A04 (N819551)	Wt
*At3g50060 (*252193*)*	MYB77	Exon	SALK_067655 (N662814)	Wt
*At4g21870 (*254384*)*	HSP26.5-P	Exon	SAIL_1284_H05 (N879227)	Wt
*At4g01250 (*255568*)*	WRKY22	Intron	SALK_047120 (N664590)	Wt
*At1g21910 (*260856*)*	DREB26	NA	NA	ND
*At1g24530 (*265028*)*	transducin /WD-40 repeat	5’-UTR	SALK_039180 (N674562)	
*At2g20670 (*265387*)*	Unknown	NA	NA	ND
*At2g26980 (*266313*)*	CIPK3	Intron	SALK_137779 (N402125)	Wt

^**a**^ Expression of common up-/down-regulated genes data were obtained from Table 2 of this study and [20].

^b^ Wt, disease response comparable to wild-type plants; S, susceptible. SAIL_737_G01 plants show increased local susceptibility to *B*. *cinerea* (Fig 4).

T-DNA insertion mutants were assayed for their disease responses at least three times.

### The *RD20* gene contributes to the plant resistance to biotic and abiotic stresses

**Fig 4:**
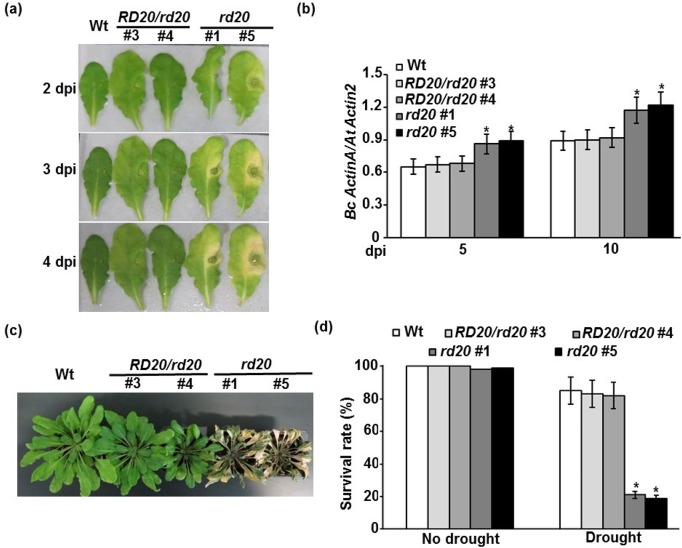
Responses of the Arabidopsis *rd20* mutant to *B*. *cinerea* infection and drought. Disease symptoms in leaves after drop-inoculation with *B*. *cinerea* (A); and fungal growth in plants after spray-inoculation with *B*. *cinerea* (B). Drought sensitivity assay on plants 10 days after stopping irrigation (C); and quantitative analysis of survival on plants continued to be not watered for 10 days and then re-irrigated for 3 days (D). In (B), qPCR amplification of *Bc ActinA* relative to the *At Actin2* gene. In (B) and (D), mean values followed by an asterisk are significantly different from the corresponding control (*P* < 0.05). All assays were repeated at least three times with similar results. Wt, wild-type; *RD20/rd20*, heterozygous line; *rd20*, homozygous *Bc ActinA*, *B*. *cinerea ActinA* gene; *At Actin2*, *Arabidopsis Actin2* gene; dpi, days post-inoculation.

The *RD20* gene was induced by *B*. *cinerea* in inoculated wild-type plants (Table 2). In order to check the function of the *RD20* gene, we isolated homozygous lines for the T-DNA insertion allele of the *RD20* gene designated *rd20* (*SAIL_737_G01*) using PCR (S2 Fig). Plants homozygous for the *rd20* allele display increased susceptibility to *B*. *cinerea* infection compared with heterozygous (*RD20/rd20*) or wild-type plants (Fig 4A). At early stages of disease, symptoms developed as local chlorosis and necrosis on inoculated leaves of the mutant *rd20*. Extending the period of inoculation to 4 days, disease symptoms developed beyond the inoculated tissues. We also determined the fungal growth *in planta*. At 5 and 10 days post-inoculation (dpi), *rd20* mutant plants exhibited more fungal biomass than the other genotypes, as assessed by accumulation of *B*. *cinerea ActinA* relative to *At Actin2* (Fig 4B).

To characterize the performance of *rd20* plants under drought stress, 3-week-old seedlings grown in soil were treated with no water to induce drought stress for additional 10 days. We noticed that the wilting levels of *rd20* mutant plants were more obvious than those of the wild-type or *RD20/rd20* plants (Fig 4C). Only 20% of *rd20* plants survived, whereas the corresponding survival rates were 82–85% for wild-type and heterozygous plants after 3 days of rewatering preceded by 10 days of water-deficit stress treatment (Fig 4D). Seedlings of all genotypes showed no death when water was applied. Altogether, this suggests that *RD20* plays an important role in plant defense to *B*. *cinerea* infection and drought stress.

### Regulation of differentially expressed genes through electrophilic oxylipin

**Fig 5:**
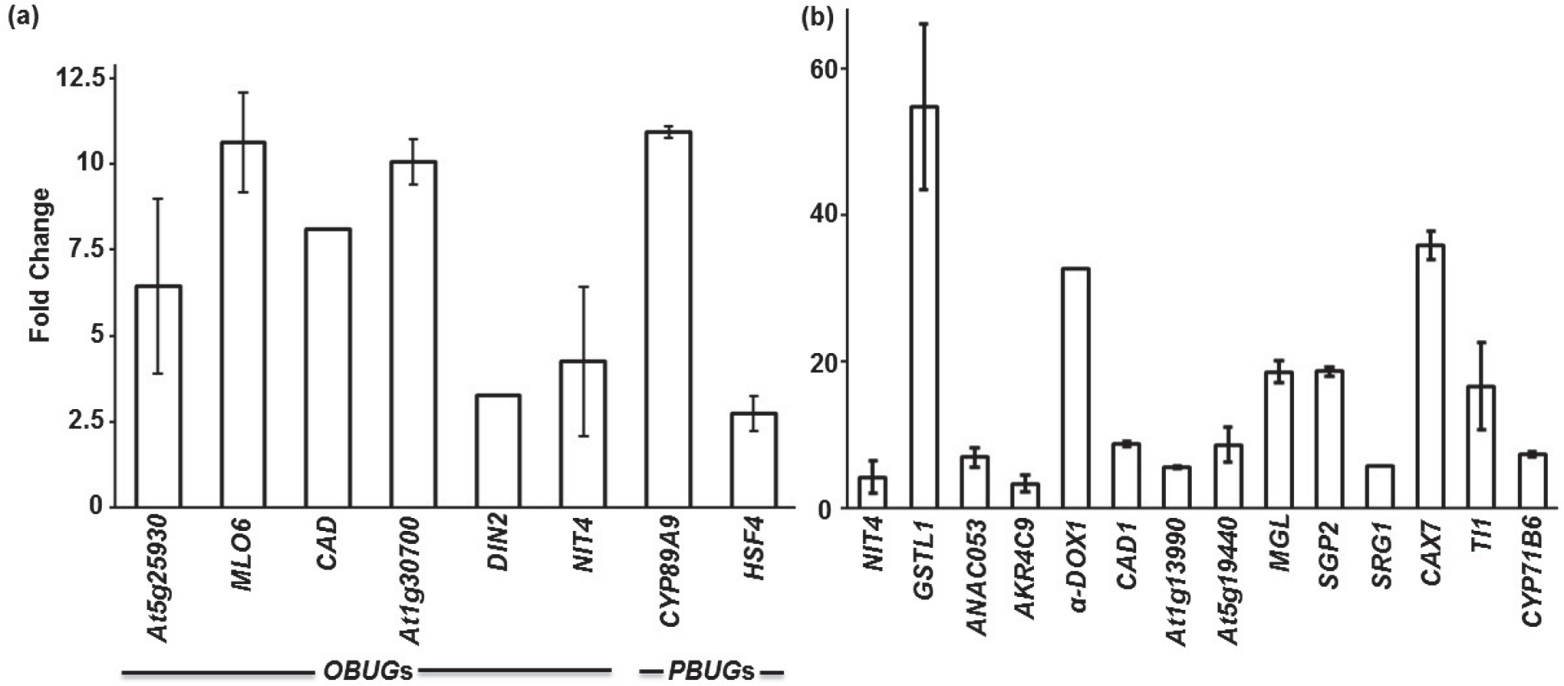
Expression of *OBUG*s/*PBUG*s and abiotic stress-regulated genes in response to *B*. *cinerea*. Relative expression levels obtained through qRT-PCR for common *OBUG*s or *PBUG* and abiotic stress-upregulated genes (A); and *OBUG*s/*PBUG*s and abiotic stress-upregulated genes (B) after infection with *B*. *cinerea* at 18 hpi. Expression of *B*. *cinerea*-inducible genes was quantitated relative to control conditions (no infection), and corrected for expression of the control gene (β-actin). Error bars for qRT-PCR values are the standard deviations (n ≥ 3). In (a) and (b), data were obtained from Tables 4 and 5, respectively. hpi, hours post inoculation; *At Actin2*, Arabidopsis *Actin2* gene.

All oxylipins, 12-oxo-phytodienoic acid (OPDA), phytoprostane A_1_ (PPA_1_) and jasmonate (JA) are regulators of stress responses [11, 27, 28]. The cyclopentenones, OPDA and PPA_1,_ activate gene expression independently from the cyclopentanone, JA. We investigated whether the regulation of OPDA or PPA_1_ respondents [11, 27] was also regulated by *B*. *cinerea*, heat, salinity and osmotic stress. Previously, it was shown that the OPDA/*B*. *cinerea* upregulated genes (*OBUG*s), *DREB2A*, *REF*, *UGT73B5*, *HSP17*.*4* and *PDR12*, and PPA_1_/*B*. *cinerea* upregulated genes (*PBUG*s), *GSTU25*, *GSTU4*, *PDR12* and *ELI3-2*, were also induced by cold, drought or oxidative stress [20]. Except of *GSTU25*, the rest of the commonly expressed genes were also upregulated by osmotic stress (Table 4). Conversely, *HSP17*.*4* was induced by salinity as well, suggesting that plant responses to osmotic stress can share common respondents with *OBUG*s and *PBUG*s and other abiotic stresses. Some of the *OBUG*s (*At5g25930*, *MLO6*, *At3g04640*, *At1g30700* and *NIT4*) and the *PBUG* (*GSTU25*) were not regulated by any of the tested abiotic stress treatments; while others such as *CAD* and *DIN2* (*OBUG*s), and *CYP89A9* and *HSF4* (*PBUG*s) were induced by salinity and/or osmotic stress (Table 4). By contrast, no *OBUG* or *PBUG* was regulated by heat treatment. The results obtained from microarrays data for *OBUG*s or *PBUG*s were confirmed by qRT-PCR analysis in response to *B*. *cinerea* infection (Fig 5A). In general, our analysis revealed that some of the OPDA- or PPA_1_-regulated genes were specifically regulated by *B*. *cinerea* (Table 4; Fig 5A); or by a particular abiotic stress (S4 Table), others were regulated by *B*. *cinerea* and abiotic stresses simultaneously (Table 4; Fig 5A).

**Table 4:** Regulation of genes by OPDA or PPA_1_ treatment, *B*. *cinerea* infection, heat, salinity and osmotic stress.

Description	Gene locus	Normalized Fold induction^a^		
		OPDA/PPA_1_ ^b^	*B*. *cinerea* ^c^	Abiotic stress^d^
***OBUG*s**		**OPDA**		
Receptor-related protein kinase like	*At5g25930*	7.1	4.6	
DRE-binding protein (DREB2A)	*At5g05410*	4.4	3.4	Os
Mildew resistance locus O6 (MLO6)	*At1g61560*	3.9	4.2	
Gly-rich protein	*At3g04640*	3.4	8.1	
Rubber elongation factor (REF)	*At1g67360*	2.0	3.5	Os,S
UDP-glucose transferase (UGT73B5)	*At2g15480*	6.7	3.1	Os
Cinnamyl-alcohol dehydrogenase (CAD)	*At1g09500*	7.2	17.5	Os,S
Class I heat-shock protein(HSP17.4)	*At3g46230*	12.4	3.3	Os,S
FAD-linked oxidoreductase family	*At1g30700*	7.9	16.5	
ABC transporter (PDR12)	*At1g15520*	18.7	22.6	Os
β-glucosidase 30; Dark inducible 2 (DIN2)	*At3g60140*	3.1	18.3	Os
Nitrilase 4 (NIT4)	*At5g22300*	3.9	4	
***PBUG*s**		**PPA**_**1**_		
CYP89A9	*At3g03470*	3.1	5.9	Os,S
GSTU25	*At1g17180*	17	10.8	
GST22/GSTU4	*At2g29460*	3.7	9.3	Os
PDR12	*At1g15520*	24.5	22.6	Os
HSF4	*At4g36990*	12.3	4.2	Os,S
ELI3-2	*At4g37990*	15	75.2	Os
Cyclin, putative	*At1g44110*	-4.4	-3.1	Os
SYP111	*At1g08560*	-4.0	-3.6	
ACT11	*At3g12110*	-3.6	-4.2	Os

^**a**^ Normalized fold induction = normalized OPDA/PPA_1_ treatment, *B*. *cinerea* inoculation or abiotic stress / normalized no OPDA/PPA_1_ treatment, no *B*. *cinerea* inoculation or no abiotic stress. Data set on at least twofold induction after treatment/inoculation.

^**b**^ OPDA-upregulated genes data were obtained from [27] at 3 hpt. PPA_1_-upregulated genes data were obtained from [11] at 4 hpt.

^**c**^ *B*. *cinerea*-upregulated genes data were obtained from [20] at 18 hpi.

^**d**^ Heat-, salt- or osmotic stress-upregulated genes data were obtained from this study at 24 hpt.

-, downregulation.

In addition, we found about 59% of the induced genes by OPDA and PPA_1_, and dependent on *TGA2/5/6* transcription factors, were also induced by *B*. *cinerea* [20]. The genes upregulated by OPDA and PPA_1_ treatments and by *B*. *cinerea*were called *OBUG*/*PBUG*s. The microarray study revealed that the genes *NIT4*, *GSTL1* and *At1g33590* (Leucine-rich repeat disease resistance protein), containing a TGA motif (TGACG) in their promoters (in the first 500 bp upstream of the start codon) were induced by *B*. *cinerea* (Table 5). The TGA motifs are potential binding sites for TGA transcription factors [11, 29]. The array results for these genes were confirmed by qRT-PCR upon infection with *B*. *cinerea* at 18 hpi (Fig 5B). Then, we identified TGA dependent-*OBUG*/*PBUG*s inducible by the three types of abiotic stresses tested in this study. Nine of the induced genes containing TGA motif in their promoters were osmotic stress-induced; six were salt-induced; and only one was heat-induced (Table 5). At 18 hpi with *B*. *cinerea*, the transcriptional analysis of the latter genes was also confirmed by qRT-PCR (Fig 5B). This suggests that the necrotrophic fungus *B*. *cinerea* and osmotic stress affect the regulation of OPDA and PPA_1_ *in planta*. On the other hand, we found that plants stressed with salt and osmotic stresses, but not heat, change the profiles of *OBUG*/*PBUG*s independently from TGA transcription factor (Table 5). Our qRT-PCR analysis showed that *B*. *cinerea* also induced these genes (Fig 5B). In addition, other upregulated respondents by OPDA and PPA_1_ treatments were upregulated by salt and osmotic stress, regardless of their regulation by *B*. *cinerea* infection (S4 Table). We also found an important overlapping in the regulation of *B*. *cinerea* and osmotic stress in plant defense system, and to lesser extent between *B*. *cinerea* and salt, affecting the cyclopentenone pathway TGA-dependent. Consequently, we conclude that there might be a unique gene regulation programing by OPDA and PPA_1_ that can be induced either by *B*. *cinerea*, abiotic stress, or in combinations.

**Table 5:** Upregulated genes by *OBUG*s and *PBUG*s, and abiotic stresses dependent on *TGA2/5/6*.

Arrayelement	Gene locus	Description^a^	TGACG^b^	Abiotic stress^c^
***OBUG/PBUG***				
*249942*	*At5g22300*	Nitrilase 4 (NIT4)	+	
*250983*	*At5g02780*	Glutathione transferase lambda 1 (GSTL1)	+	
*245768*	*At1g33590*	Disease resistance LRR protein-related	+	
*266995*	*At2g34500*	CYP710A1	+	Os
*258921*	*At3g10500*	NAC domain containing protein 53 (ANAC053)	+	Os
*267168*	*At2g37770*	Aldo/keto reductase family protein (AKR4C9)	+	Os,S
*250948*	*At5g03490*	UDP-glucoronosyl/UDP-glucosyl transferase	+	Os,S
*258957*	*At3g01420*	Alpha-dioxygenase 1 (α-DOX1)	+	Os
*259911*	*At1g72680*	Cinnamyl alcohol dehydrogenase 1 (CAD1)	+	Os,S
*262607*	*At1g13990*	Expressed protein	+	Os,S
*249860*	*At5g22860*	Ser carboxypeptidase S28 family protein	+	H,Os,S
*263517*	*At2g21620*	Responsive to dessication 2 (RD2)	+	Os,S
*250054*	*At5g17860*	Calcium exchanger 7 (CAX7)	-	Os
*258277*	*At3g26830*	Phytoalexin deficient 3 (PAD3)	-	Os
*246042*	*At5g19440*	Alcohol dehydrogenase	-	Os,S
*261957*	*At1g64660*	Catalytic/methionine gamma-lyase (MGL)	-	Os,S
*257951*	*At3g21700*	Small GTPase (SGP2)	-	Os
*262482*	*At1g17020*	Senescence-related gene 1 (SRG1); oxidoreductase	-	Os
*260551*	*At2g43510*	Trypsin inhibitor protein (TI1)	-	Os
*266000*	*At2g24180*	CYP71B6	-	Os

^**a**^ Normalized fold induction of genes by PPA_1_ and OPDA (75 μM) at 4 hpt and *B*. *cinerea* at 18 hpi at least twofold in Arabidopsis wild-type plants relative to controls but no induction in *tga2/5/6*. *OBUG*- and *PBUG*-induced genes data were obtained from [20].

^**b**^ Promoters of genes containing a TGA motif (TGACG) in the first 500 bp upstream of the start codon were obtained from [11].

^**c**^ Normalized fold induction of genes by heat, salinity or osmotic stresses of at least twofold in Arabidopsis wild-type plants relative to controls (S2 Table). Abiotic stress-induced genes data were obtained from this study at 24 hpt.

## Discussion

Plant responses to simultaneous biotic and abiotic stresses are mostly controlled by different hormonal and non-hormonal signaling pathways that may interact with each other, through the activation of transcription factors, effector proteins and secondary metabolites [3, 5, 18, 30–32]. Plants that were exposed to a given biotic stress are often more susceptible to abiotic stresses and *vice versa* [33, 34]. To elucidate the relationship between the two types of stresses, many reports have focused on the regulatory crosstalk between biotic and abiotic stress responses. Expression profiling of plant response to one type of stress-*B*. *cinerea* infection or abiotic stress treatment- has been well-documented [21, 25, 35, 36]. In addition, transcriptome analysis of Arabidopsis, rice, tobacco (*Nicotiana tabacum*) and cotton (*Gossypium hirsutum* L.) revealed crosstalk of responsive genes to various abiotic stresses [37–40]. Combinations of different biotic and abiotic stresses have allowed the identification of candidate genes involved in broad resistance [41]. A recent transcriptome analysis showed shared regulated genes when Arabidopsis plants were infected with *B*. *cinerea* or treated with cold, drought or oxidative stress [20]. Here, we extended the comparative microarray analysis, obtained from Arabidopsis public databases, to include *B*. *cinerea*, heat, salinity and osmotic stresses. We identified up- and down-regulated genes after treatments with an individual stress, or upon a combination of biotic and abiotic stresses. In response to *B*. *cinerea*, approximately 7% of genes were induced and 5% were repressed across the whole *Arabidopsis* transcriptome [20]. The transcript levels of 153 and 799 genes increased more than twofold after heat and high salinity treatments, respectively, compared with the control genes; but 507 and 872 genes had impaired transcript levels of the transcripts for the same treatments (Fig 1). The largest number of genes up- or down-regulated by a specific stress corresponded to osmotic stress with 1695 or 2210 genes, respectively. Previously, it was also found that the number of genes induced by salt stress in cotton was greater than in any other type of abiotic stress, particularly cold, pH or osmotic stress [40]. Based on the molecular and functional classifications and comparisons, some abiotic stress-regulated genes have been classified as genes, with known functions such as transcription regulators, scavengersor ion transporters [39, 40]; yet many remain unknown. We closely looked to the relationship between gene regulation in response to *B*. *cinerea* infection and in response to heat, salinity or osmotic stresses. We found that osmotic stress and *B*. *cinerea* shared the highest number of regulated genes; while heat and *B*. *cinerea* shared the least. Although a significant number of differentially expressed genes were regulated under specific stresses; others were also co-regulated by a combination of different stresses. We observed strong correlations of stress-associated genes and found that 13 stress-inducible genes and 29 stress-repressible genes have responded to all four types of stresses (Fig 2). We expanded the analysis to include other transcriptome studies and we noticed that there were large fluctuations in the percentage of co-regulated genes (up- or down-regulated) between biotic (*B*. *cinerea*), and abiotic stresses, as shown in Table 1 as 58% cold, 12.9% drought, 17.2% oxidative stress, 10.1% heat, 37.9% salinity, and 89% osmotic stress (Table 1).

Microarray transcriptional profiling demonstrated that *lecithin*:*cholesterol acyltransferase 3* (*LCAT3*) gene, encoding for phospholipase A_1_ (PLA1) enzyme [42], was upregulated after infection with *B*. *cinerea* or treatment with heat, 150 mM NaCl or 300 mM mannitol (Fig 3). In addition, the expression of Arabidopsis *LCAT3* in yeast resulted in a doubled content of the triacylglycerol [43]. The *Defective in Anther Dehiscence1* (*DAD1*) is another PLA1 involved in basal JA production and resistance to *B*. *cinerea* [44]. The putative transposable element gene *At2g06890* was induced by the four types of stresses tested, suggesting a potential role of *LCAT3* and *At2g06890* in plant response to environmental stress. Our analysis also showed that the transcript levels of *ESE3*, an ERF/AP2 transcription factor, were impaired in plants sprayed with *B*. *cinerea* or treated with NaCl; which seems to be in disagreement with a previous study reporting an induction of this gene by salt stress [45]. This discordance could be attributed to the different plant growth conditions and NaCl concentrations.

It is noteworthy to mention that only three genes were commonly induced by the seven types of stresses (six types of abiotic stresses and one type of biotic stress; *B*. *cinerea*) and 12 genes were repressed (Table 1); suggesting extensive overlapped responses to these genes to different types of biotic and abiotic stresses. Arabidopsis *Responsive to Dehydration20* (*RD20*; *At2g33380*), also known as *Caleosin3* (*CLO3*), was among the common induced genes in response to biotic and abiotic stresses (Table 3). The *RD20/CLO3* gene encodes a Ca^+^-binding protein, was induced by ABA, drought and high salinity [46–48]. The induction of Arabidopsis *RD20* [20] and the sensitivity of its mutant to drought in Col-0 ecotype (Fig 4) confirmed previous data in Wassilewskija (Ws-4) ecotype after drought stress treatment [46]. These findings demonstrate that *RD20* is involved in the response of Arabidopsis to abiotic stresses. It was reported that *RD20* was strongly induced by the reactive oxygen species (ROS)-inducing herbicide, paraquat [49]. In addition, the Arabidopsis *rd20* mutants showed enhanced sensitivity to oxidative stress [50]. Because enhanced generation of ROS was found to accompany infections caused by necrotrophic pathogens [51], we hypothesize that *RD20* may confer resistance against *B*. *cinerea*. First, we found that the transcription of the stress-induced caleosin gene *RD20* was upregulated by *B*. *cinerea* (Table 2) and by other pathogens [20, 46, 52]. Second, functional analysis on *rd20* mutants demonstrated that *RD20* plays a significant role in plant defense against the necrotrophic fungi *B*. *cinerea* (Fig 4) and *Alternaria brassicicola* [53] but not the hemibiotroph *P*. *syringae* [46], suggesting an involvement of the caleosin RD20 in Arabidopsis responses to necrotrophic pathogens. Taken together, these findings reveal a novel role for *RD20*/*CLO3* in regulating plant stress response.

It has been reported that *At5g25930* (LRR receptor-related kinase protein) and *MLO6* (Mildew Resistance Locus O6), *At1g30700* (FAD-linked oxidoreductase) and *NIT4* (Nitrilase4) were induced after inoculation with *B*. *cinerea* or other pathogens [27]; supporting our results here about the involvement of these genes in the biotic stress signaling through OPDA. Our analysis showed that *CAD*, involved in lignin biosynthesis, and *DIN2* (glycosyl hydrolase), involved in cellular sugar response, were induced by pathogen challenges, abiotic stresses and OPDA treatments [20, 54, 55], suggesting that modifications in cell wall properties and functions occur during plant responses to stress. On the other hand, the induction of *CYP89A9* and the heat shock factor, *HSF4*, by *B*. *cinerea*, high salt or osmotic stress (Table 4; Fig 5) is an evidence that these genes are involved in pathogen and abiotic stress signaling [56], mediated by the electrophilic oxylipin PPA_1_ [11]. In the same report [56] as well as in others [6], the *B*. *cinerea*-inducible genes, *At5g25930*, *HSF4* and *BIK1*-whose mutant showed increased susceptibility to *B*. *cinerea*-, suggest potential roles in plant stress response/defense. Deeper investigation about the role of these genes in response to environmental stresses through cyclopentenones is required.

A recent transcriptomic and metabolomic analyses on copper-stressed brown algae (*Ectocarpus siliculosus*) showed accumulation of oxylipin compounds and shared responses with oxidative stress and NaCl treatments [57]. These findings are in agreement with our observations (Table 4) and a previous study on kelp [58]. Moreover, *Methionine gamma lyase* (*MGL*) gene, involved in methionine homeostasis [59], was upregulated by oxylipin cyclopentenones, *B*. *cinerea* infection, salinity and osmotic stress (Table 5; Fig 5), suggesting that MGL may regulate methionine metabolism under combinatory conditions of different stresses. By contrast, *azelain acid-induced1* (*AZI1*) gene, involved in priming defense in systemic plant immunity [60], was downregulated in leaves treated with *B*. *cinerea* or abiotic stresses (Table 2). In a recent transcriptome study on Arabidopsis leaves exposed to both drought and beet cyst nematode (*Heterodera schachtii*) revealed that *MGL* was induced and *AZI1* was repressed [18]. In the same report, transgenic lines overexpressing *MGL* and *AZI1* confer resistance to nematodes and sensitivity to drought, respectively; suggesting that MGL and AZI1 may play a key role in plant response to biotic and abiotic stresses.

On the other hand, three membrane-associated transcription factors (MTFs), bZIP28, bZIP60 and NAC089, play important roles in the regulation of plant cell death (PCD) under stressful conditions in Arabidopsis [61, 62]. *NAC089* has been reported as inducible by the endoplasmic reticulum (ER) stress and controlled by bZIP28 and bZIP60; suggesting that NAC089 regulates the downstream targets NAC094, MC5 and BCL-2-associated athanogene (BAG6), involved in PCD during plant ER stress response. Similarly, the identification of genes encoding NAC053, BAG6, WRKY22 and WRKY47 transcription factors suggests significant roles of these genes in the regulation of PCD-related genes through enzymatic or non-enzymatic pathways. The investigation of the function of the regulated genes and their downstream targets under multiple stresses is underway.

## Conclusion

Accumulating databases in Arabidopsis genome research have enabled integrated genome-wide studies to be performed to dissect plant responses to multiple diseases and variable biotic and abiotic stress conditions. Based on public databases relevant to our purposes, we tried to perform an analytic process to explore transcriptome data to predict consistent/inconsistent patterns and/or systematic interactions between various biotic and abiotic stresses. Our goal was to apply predictive data mining toward better comprehension of the complex biological systems that control plant/environment interactions and to provide valuable insights into gene function/dynamic relationships at the molecular levels. Many genes identified in this study could serve as general markers of common responses to biotic and abiotic stresses, and in some cases as responses mediated by oxylipin cyclopentenones. Along with the functional analysis, the identification of common regulators of plant responses to environmental constraints should enlighten the road of genetic engineering and serve breeding programs to develop broad-spectrum stress-tolerant crops. Future research to dissect specific functions of stress-involved components and to map all implicated elements in stress signal transduction pathways should be a priority focus. Follow-up studies benefiting from available resources and upcoming technical and methodological advancements in basic and applied researches should offer valuable tools in complement to the assessment of transcriptome analysis that would reflect, as faithfully as possible, the *in vivo* complexity of biological systems against multiple, simultaneous environmental conditions.

## Supporting Information

S1 Fig Functional classes of abiotic stress-regulated genes. (A) heat-, (C) salinity- and (E) osmotic stress-upregulated genes; and (B) heat-, (D) salinity- and (F) osmotic stress-downregulated genes at 24 hpt compared with 0 hpt of wild-type leaf tissues. Error bars are SD. GO categories that are significantly over- or under-represented at *P* < 0.05, are in black text. Normalized frequency of genes to the number of genes on the microarray chip was determined as described [63].
(PDF)

S2 Fig Genotyping of the *rd20* insertion mutants using PCR. M, marker; LP/RP, primer to the left/right of the T-DNA insertion; LB, T-DNA left border sequence was used for PCR amplification of plant flanking sequences; GSP, gene-specific primer. The asterisk represents homozygous lines used for further disease assays.
(PDF)

S1 Table List of qRT-PCR primers (sequence 5’ to 3’) used in this study. (PDF)

S2 Table Expression levels and fold induction of all (A) heat-, (C) salinity-, and (E) osmotic stress-upregulated genes; or repression of all (B) heat-, (D) salinity-, and (F) osmotic stress-downregulated genes, selected from wild-type samples. (XLSX)

S3 Table List of probe sets/array elements and locus identifiers corresponding to genes that are induced (A-C) or repressed (D-F) by *B*. *cinerea* inoculation and heat (A, D), salinity (B, E), and osmotic stress (C, F). (XLSX)

S4 Table Regulation of genes by PPA_1_ or OPDA treatment and abiotic stress. (PDF)
