# Supplementary figures and images for: Identification of Arabidopsis Candidate Genes in Response to Biotic and Abiotic Stresses Using Comparative Microarrays
Source: PLoS One. 2015 May 1;10(5):e0125666. doi: 10.1371/journal.pone.0125666 (PMC4416716; doi:10.1371/journal.pone.0125666)

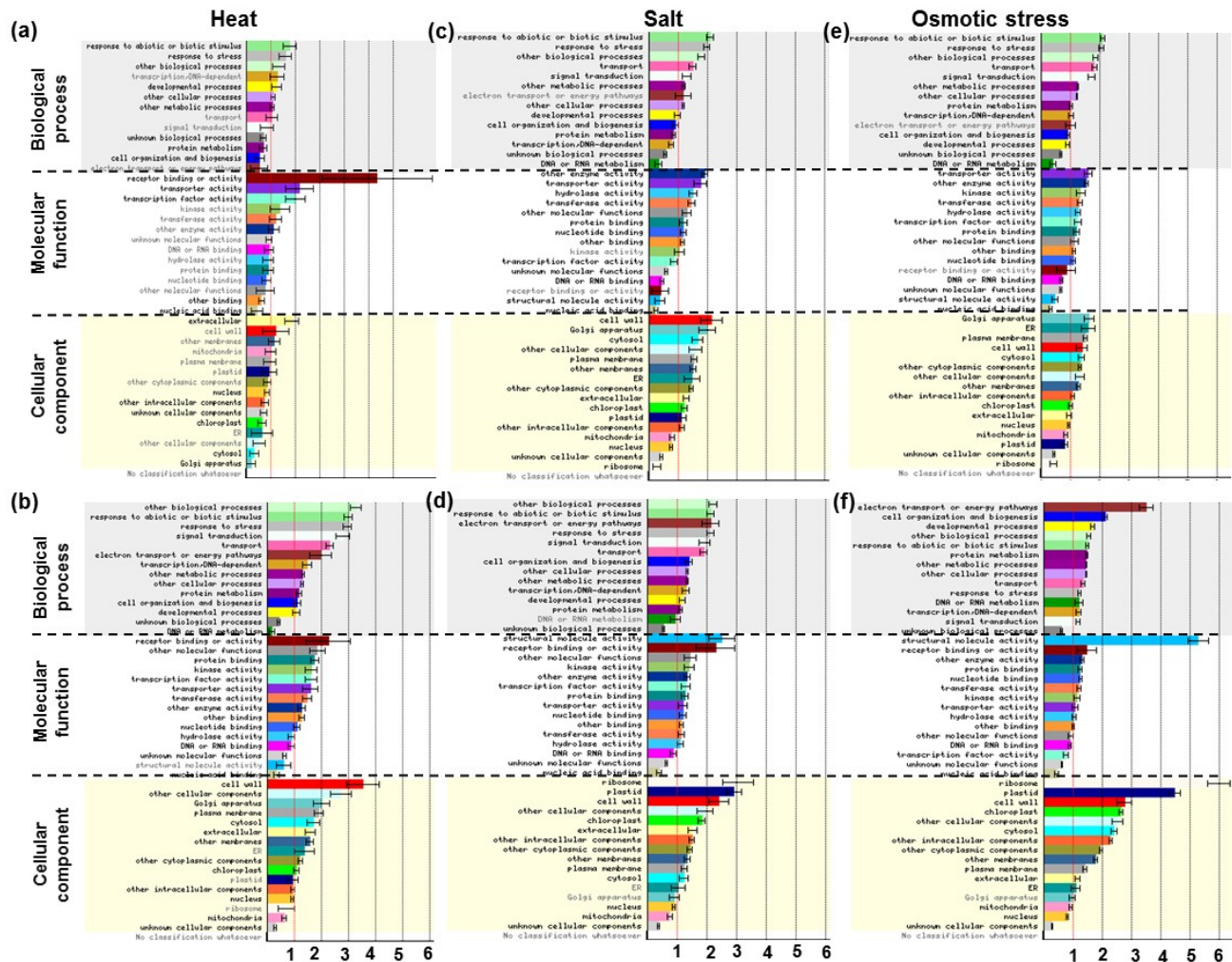

Supplement: S1 Fig — (A) heat-, (C) salinity- and (E) osmotic stress-upregulated genes; and (B) heat-, (D) salinity- and (F) osmotic stress-downregulated genes at 24 hpt compared with 0 hpt of wild-type leaf tissues. Error bars are SD. GO categories that are significantly over- or under-represented at P < 0.05, are in black text. Normalized frequency of genes to the number of genes on the microarray chip was determined as described [63]. (PDF) [file pone.0125666.s001.pdf]

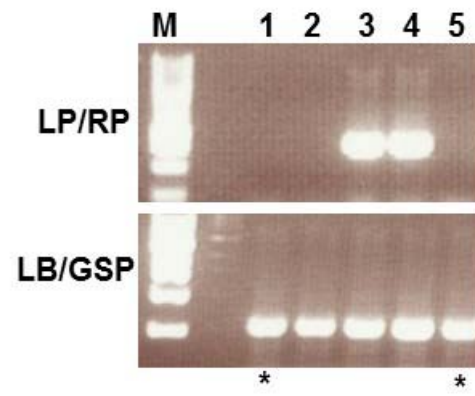

Supplement: S2 Fig — M, marker; LP/RP, primer to the left/right of the T-DNA insertion; LB, T-DNA left border sequence was used for PCR amplification of plant flanking sequences; GSP, gene-specific primer. The asterisk represents homozygous lines used for further disease assays. (PDF) [file pone.0125666.s002.pdf]
