# Supplementary material for: Identification of Arabidopsis Candidate Genes in Response to Biotic and Abiotic Stresses Using Comparative Microarrays
Source: PLoS One. 2015 May 1;10(5):e0125666. doi: 10.1371/journal.pone.0125666 (PMC4416716; doi:10.1371/journal.pone.0125666)
Supplement: S1 Table — (PDF) [file pone.0125666.s003.pdf]

Table S1. List of primers (Sequence 5' to 3') used in this study.

Description	Left primer sequence	Right primer sequence
AtActin2	GTCGTACAACCGGTATTGTGCTG	CCTCTCTCTGTAAGGATCTTCATGAG
LCAT3	GATGTTTGTTATGGCACAGAGACTT	GGACAGTTCCATCTCCATCTACATA
At2g06890	GAGTTGTACTAATGTGGCAAGTGAA	ACTGTCATTAAGCCACTAAAGATCG
BAG6	GAAGCTGTACTTCCAGTTCGTTCTA	ACCCAAGAGAGCTTCTATATTCCTG
ESE3	AGAGCTCGTACTAATTTCCCATACA	CATGTAGCATTTGTGGAGTTTAGC
At5g25930	GAGAAGGAGTTTATTGCTGAAGTTG	AGCTTTGAATCTTCCCTTGAGATAC
MLO6	TGCAGCTACGTTACTCTTCCTCTAT	ACTCTTTAACGCTGTTGCTACTCTC
REF	TTGGTTATCTTCCGTTGGTTCCTGT	CTTTCTTTCCAGCCGTATCCCCTCC
CAD	GGTAGCTGTGATTGTGGAACTTATG	CTTTACATGAGCTAGAGCAACATCTCT
At1g30700	GTAGTAAACGGCACTGTTTCATCTC	CGGTTTAATATCGACAGTAGAGTTGTC
MLO6	CAATCTCGTGGTCAAGATTAATACC	TAGCAAGAAGTTCGTCTATGAGATCC
DIN2	CAATCTCGTGGTCAAGATTAATACC	AAGAAGTTCGTCTATGAGATCCTTG
NIT4	CATCAAATCTTCCCTGAGATTGAC	CGGGAGTATCGTAGAAGACTGTAGA
CYP89A9	ATCTCCGACGCTTTAGATCATTTAC	TCGATCTCTCTGATCTCTTCTTTTC
HSF4	GCTAGTTGATGATCATAGCACAGAC	GAAGATCTTTAGCAAACTCTGCTGT
GSTL1	GATTACAAGAAACCTCAAGGGTCTG	GCAGGATTAACTTTCTCCTTTAACC
NTL4	GACGAAGAACTCGTTCGTTACTATC	GGCTCAGATTTGTATACATCGGTAA
AKR4C9	CATGAGACACTAAGCCCTTACAAGT	AGAGAGAGAAACCTAGCTCCCATTA
CAD1	ATGAGTTCTTCAGAGAGTGTGGAAA	TAACAGACCTGCGAGTGATAGTATG
At1g13990	CAGGCTAGACCCAAAACTAAATTC	GGTCACAGTATCTAACAGCCTCATT
At5g19440	TTAACATTCTACGTGAGCTTTACCC	CGTTTTATCCTTGGACACTTGATAC
MGL	GACATGTAACATAACCACCTCCTTC	GACTCAAAGTAGAGAACCTGTGTCC
SGP2	GACTCTGTTGCCATTCTCTTTATGT	CGTCTGATTAGACTTCCTAGCTTGT
SRG1	ATCAGAAACTAGATTGGGCAGACTT	TATCTCTAAAGGGAAGAGGTAGCTTG
CAX7	CCTGGTTGTTTGTTCTGTTCTACTT	TAGGAGACAGCTTCAAAACCTTAGA
TI1	CTATCGTTTCCATCTTCGTTGTCT	GTATTCTTTCAAGCACTCGTTACCT
CYP71B6	TACTCGTCCAAAGCTATCTATCACC	AGTTTCCGCACATCTCTGTAGTAAT
