# Supplementary material for: Identification of Arabidopsis Candidate Genes in Response to Biotic and Abiotic Stresses Using Comparative Microarrays
Source: PLoS One. 2015 May 1;10(5):e0125666. doi: 10.1371/journal.pone.0125666 (PMC4416716; doi:10.1371/journal.pone.0125666)
Supplement: S4 Table — (PDF) [file pone.0125666.s006.pdf]

Table S4. Regulation of genes by PPA_1 and OPDA treatment and abiotic stress.

Description	Gene locus	Normalized fold induction*		
		PPA_1§	OPDA§	Abiotic stress‡
17.6-kD heat-shock protein (AA 1-156)	*At1g53540*	N	13.5	S
Class II heat-shock protein	*At5g12020*	N	12.5	S
Heat-shock protein 17.6A (AT-HSP17.6A)	*At5g12030*	N	13.2	Os,S
Heat-shock protein family	*At5g37670*	N	3.0	H,Os,S
Heat-shock protein family, putative	*At2g20560*	N	7.2	Os,S
Ser/Thr kinase-like protein	*At4g23190*	N	-3.3	H
Copper/zinc superoxide dismutase (CSD2)	*At2g28190*	N	-2.5	Os,S
Copper Chaperine for SOD1 (CCS)	*At1g12520*	N	-2.5	Os,S
UDP-glucoronosyl/UDP-glucose transferase	*At4g01070*	4.2	N	Os
UDP-glucoronosyl/UDP-glucose transferase	*At2g30140*	3.7	N	Os
Heat shock protein 70 (HSP70)	*At3g12580*	5.4	N	Os,S
β-Ig-H3 domain–containing protein/fasciclin domain–containing protein	At3g11700	-5.1	N	Os
Tubulin β-8 chain (TUBB8)	*At5g23860*	-3.8	N	Os
Cyclin delta-3 (CYCD3)	*At4g34160*	-3.5	N	Os
Kinesin motor family protein (NACK1)	*At1g18370*	-3.2	N	Os
Cell division control protein, putative	*At1g76540*	-3.1	N	Os
Endo-xyloglucan transferase (TCH4)	*At5g57560*	-5.1	N	H
Expansin B3 (EXPB3)	*At4g28250*	-4.9	N	Os
Hyp-rich glycoprotein family protein	*At3g02120*	-4.9	N	Os
glycoside hydrolase family 28/polygalacturonase (pectinase) family	*At3g06770*	-4.1	N	Os
Auxin efflux carrier protein, putative	*At1g23080*	-6.8	N	Os
Auxin-responsive AUX/IAA family protein	*At4g32280*	-5.2	N	S
Auxin efflux carrier protein, putative (PIN1)	*At1g73590*	-4.3	N	Os
IAA4/AUX2-11	*At5g43700*	-3.8	N	Os
Cytochrome P450 family (CYP72A8)†	*At3g14620*	3.8	2.7	Os
AFG1-like ATPase family protein†	*At4g30490*	2.2	2.2	Os,S
Elicitor-activated gene 3 (ELI3-1)†	*At4g37980*	2.2	2.7	Os,S

*Normalized fold induction = normalized phytoprostane-A_1 (PPA_1) or 12-oxo-phytodienoic acid (OPDA) treatment and abiotic stress/normalized no PPA_1 or OPDA treatment and no abiotic stress. Except for CYP72A8, AFG1-like ATPase and *ELI3-1*, data set on at least threefold induction/repression after treatment. *CYP72A8*, *AFG1-like ATPase* and *ELI3-1* fold induction by PPA_1 and OPDA (75 µM) of at least twofold in Arabidopsis plants relative to control but no induction in *tga2/5/6* at 4 hpt (Mueller et al., 2008).

No TGA motif (TGACG) was identified in the promoters of the gene

§OPDA or PPA_1-upregulated genes data were obtained from Taki et al., (2005) at 3 hpt or Mueller et al. (2008) at 4 hpt, respectively.

‡Heat (H), salt (S) or osmotic stress (Os)-upregulated genes data were obtained from this study At 24 hpt.

N, not expressed; -, downregulation.
